# Tumor Necrosis Factor Receptor Superfamily, Member 1B Haplotypes Increase or Decrease the Risk of Inflammatory Bowel Diseases in a New Zealand Caucasian Population

**DOI:** 10.1155/2009/591704

**Published:** 2009-05-03

**Authors:** Lynnette R. Ferguson, Dug Yeo Han, Claudia Huebner, Ivonne Petermann, Murray L. Barclay, Richard B. Gearry, Alan McCulloch, Pieter S. Demmers

**Affiliations:** ^1^Discipline of Nutrition, The University of Auckland, Private Bag 92019, Auckland 1023, New Zealand; ^2^Nutrigenomics New Zealand, New Zealand; ^3^Department of Gastroenterology, Christchurch Hospital, Christchurch 8011, New Zealand; ^4^Department of Medicine, University of Otago Christchurch, New Zealand; ^5^Crop and Food Research, Mosgiel 9053, New Zealand; ^6^AgResearch Limited, Mosgiel 9053, New Zealand

## Abstract

Inflammatory bowel diseases (IBDs) comprising Crohn disease (CD) and ulcerative colitis (UC) are chronic inflammatory conditions with polygenic susceptibility. Interactions between TNF-alpha and TNF-alpha receptor play a fundamental role in inflammatory response. This study investigates the role that selected single nucleotide polymorphisms (SNPs) and haplotypes in the TNF-alpha receptor (TNSFRSF1B) gene play in the risk of IBD in a New Zealand Caucasian population. DNA samples from 388 CD, 405 UC, 27 indeterminate colitis patients, and 293 randomly selected controls, from Canterbury, New Zealand were screened for 3 common SNPs in TNSFRSF1B: rs1061622 (c.676T > C), rs1061624 (c.*1663A > G), and rs3397 (c.*1690T > C), using TaqMan technologies. Carrying the rs1061624 variant decreased the risk of UC in the left colon (OR 0.73, 95% CI = 0.54–1.00) and of being a smoker at diagnosis (OR 0.62; 95% CI = 0.40–0.96). Carrying the rs3397 variant decreased the risk of penetrating CD (OR 0.62, 95% CI = 0.40–0.95). Three marker haplotype analyses revealed highly significant differences between CD patients and control subjects (*χ* ^2^ = 29.9, df = 7, *P* = .0001) and UC cases and controls (*χ* ^2^ = 46.3, df = 7, *P* < .0001). We conclude that carrying a 3-marker haplotype in the TNSFRSF1B gene may increase (e.g., haplotype of GGC was 2.9-fold more in the CD or UCpatients) or decrease (e.g., TGT was 0.47-fold less in UC patients) the risk of IBD in a New Zealand Caucasian population.

## 1. Introduction

Inflammatory bowel diseases (IBDs), including Crohn disease (CD) and ulcerative colitis (UC), provide a good example of a disorder for which there is compelling evidence for genetically determined susceptibility that interacts with environmental factors, including microbiota and diet [1]. It is clear that there are strong ethnic differences in IBD susceptibility, and that different populations may have varying levels of genetic involvement, with distinctive risk alleles [2]. For example, although there is a very low incidence of CD in Bangladesh, the children of Bangladesh immigrants to the UK show a very high incidence of the disease [2]. As recently as 2005, it seemed that a maximum of 10–12 genetic loci could be involved in disease susceptibility [3], but whole genome association studies have changed the landscape such that a more recent meta-analysis [4] suggested involvement of more than 30 different loci. It becomes important to establish which loci are relevant to individual population groups living in specific locations.

The prevalence and incidence of IBD in New Zealand are believed to have increased substantially in recent years, although stringently collated data are not available prior to 2004 [5]. As at 1 June 2005, Gearry et al. (2006) reported a prevalence of IBD in Canterbury, New Zealand, as 308.3 per 100 000 subjects (155.2 with CD and 145 with UC per 100 000). Although we have previously associated polymorphisms in several genes including NOD1&2, TLR4, DLG5, ATG16L1, and IL23R with risk of IBD in New Zealand [6–12], these only increase the IBD risk to a small extent and cannot explain the high New Zealand disease incidence. For example, carrying the common NOD2 1007-femtdsecond mutation increased the risk of CD 4.4 fold (95% confidence interval 1.6–12) [12].

Tumor necrosis factor-alpha (TNF-*α*) is a proinflammatory cytokine that plays a central role in IBD pathogenesis [8]. An inhibitor of TNF-*α*, infliximab (IFB), is an effective treatment of moderate to severe IBD, and polymorphisms in the TNF-*α* gene not only affect the phenotype of IBD, but may also impact IFB susceptibility [8, 13]. However, TNF-*α* does not work independently in the cell, but acts through binding to two receptors, TNF receptor superfamily, member 1A (TNFRSF1A or p55/p60) and TNF receptor superfamily, member 1B (TNFRSF1B, also called TNFR, p75/p80). TNFRSF1B is the larger of these receptors, being present on many cell types, and strongly expressed on stimulated T and B lymphocytes [14]. There is evidence that it regulates the binding of TNF-*α* to TNFRSF1A, and thus may regulate the levels of TNF-*α* necessary to stimulate the action of the transcription factor, nuclear factor-kappa B (NF-kB) [15]. The gene for TNFRSF1B is located on chromosome 1p36.3-p36.2 [16–18], which coincides with the previously identified IBD susceptibility locus, IBD7 [19].

Single nucleotide polymorphisms (SNPs) in TNFRSF1B have been suggested to modulate the risk of a number of autoimmune diseases, including rheumatoid arthritis, systemic lupus erythematosus, and CD [20–24]. This study considers the role of three SNPs in TNFRSF1B Table 1 on the overall risk or phenotype of IBD in a New Zealand Caucasian population, recruited from the Canterbury region in New Zealand [5] 2006. These SNPs were previously associated with CD risk in other populations [22, 23].

## 2. Materials and Methods

### 2.1. Study Participants

The cases in this study are a random subset of the Caucasian participants of the Canterbury Inflammatory Bowel Disease Project, which has been described in detail elsewhere [5]. All subjects consented to collection of peripheral blood for DNA extraction and genotyping. Phenotypic characteristics of this cohort are described in more detail by Tarrant et al. [25]. The database included their clinical diagnosis and IBD phenotype as defined using standard diagnostic criteria and using the Montreal Classification system [26]. The Montreal classification was current as of 1 June 2005.

388 CD participants, 403 UC participants, and 27 indeterminate colitis (IC) participants were genotyped for this study, as described in Table 2. All participants self-reported European ancestry, and patients who self-reported having any Maori or other non-Caucasian ancestry are not included.

The New Zealand Caucasian controls (*n* = 293) were selected at random from the electoral roll, comprising 93% of the population over eighteen years of age in Canterbury, New Zealand [27].

### 2.2. Applied Biosystems TaqMan SNP Genotyping Assay

SNPs were genotyped using the TaqMan MGB diallelic discrimination system. Probes and oligonucleotides were obtained from Applied Biosystems using the Assay-by-Design product (listed in Table 3). For the marker c.676T > C (rs1061622) a predesigned SNP genotyping assay was available (C_8861232_20). The reactions were prepared by using 2*x* TaqMan Universal Master Mix, 40*x* SNP Genotyping Assay Mix, DNase-free water, 10 ng genomic DNA in a final volume of 5 uL per reaction. The PCR amplification was performed using the ABI Prism 7900 HT sequence-detector machine under the following conditions: 10 minutes 95°C enzyme activation followed by 40 cycles at 92°C for 15 seconds and 60°C for 1 minute (annealing/extension). The allelic discrimination results were determined after the amplification by performing an endpoint read. To estimate genotype accuracy approximately 5% of the samples, that is, 70 cases were genotyped in duplicate or triplicate for each of the markers. No conflicted duplicate genotypes were detected.

#### 2.2.1. Statistical Analysis

The allelic trend test [26] and Fisher's exact genotypic test were used to compare case and control allele frequencies. An exact test was used to test for departures from Hardy-Weinberg equilibrium (HWE) in the case and the control samples [27]. Allelic odds ratios were calculated and confidence intervals for the allelic odds ratio were also calculated under the assumption of HWE in the cases and the control groups. Logistic regression analysis was used to examine the relationship between genotypes and disease of CD, UC, and IBD using SAS (V9.1 SAS Institute., Cary, NC, USA). Haplotype analysis was carried out using *haplo.stats* in R [28] to test associations between haplotypes and traits. We also performed an exploratory analysis of allele frequency differences between controls and patient subgroups using the clinical characteristics given in Table 2.

## 3. Results

No significant deviation from Hardy-Weinberg Equilibrium was found in the control group for all three SNPs. For each of the polymorphisms studied, the risks of carrying the variant was compared CD and UC patients with the control group, as shown in Tables 4 and 5. There was no significant difference in the allele frequencies of the TNFRSF1B rs1061622, rs1061624, and rs3397 variants between CD or UC patients and healthy control subjects.

Individuals carrying the rs1061622 variant showed no significant differences from those carrying the reference allele (the G allele), for overall risk of IBD or for any phenotypic class. No significant differences found any of the age at first diagnosis, any location of CD and UC, or CD behavior. Having any relatives with IBD, bowel resection, smoke at diagnosis, ever used immunomodulators, or any EIMs were not significanty different between CD or UC patients and controls.

For the rs1061624 variant, those individuals carrying the A allele had a decreased probability of developing UC located in the left colon (OR = 0.73, 95% CI = 0.54–1.00, *P* = .05). Carrying the A allele also reduced the probability of UC patients having smoked at diagnosis (OR = 0.62, 95% CI = 0.40–0.96, *P* = .03) but showed no statistically significant effects in patterns of CD disease risk.

Those individuals carrying the variant TNFRSF1B rs3397 C allele showed a statistically significant reduction in the probability of developing CD with penetrating behavior, as compared with those carrying the T allele (OR = 0.62, 95% CI = 0.40–0.95, *P* = .03). No other phenotypes showed significant effects in patterns of disease risk of CD. For UC patients, those individuals carrying the variant rs3397 C allele showed no statistically significant effects in patterns of IBD (Table 4).

### 3.1. Haplotype Analysis

Table 6 summarizes the results of haplotype analysis of the three TNFRSF1B SNPs (rs1061622, rs1061624, and rs3397). Haplotype frequencies were estimated, and association analyses were performed with respect to CD and UC in New Zealand IBD patients and Caucasian controls. A score for each haplotype (Hap-score) was calculated and *P*-value also calculated for the significance of each Hap-score. A positive hap-score implies that the haplotype occurs more frequently in the CD or UC case group than control subjects. A global *P*-value tests the overall association between haplotypes and the response.

There was a significant difference between these haplotypes for CD patients and control subjects (*χ* ^2^ = 29.9, df = 7, *P* = .0001). In the three SNPs (rs1061622, rs1061624, and rs3397) in CD, the frequencies of haplotypes of GGC (9.1% for case versus 3.1% for control group, *P* = .005) and TAT (17.2% versus 6.4%, *P* = .001) were significantly different, with haplotypes GGC and TAT occurring more in CD cases than the control group. TAC (21.0% versus 27.7%, *P* = .06) and TGT (14.5% versus 23.6%, *P* = .06) were marginally significantly different between case and control groups, occurring less frequently in the cases. Haplotype TAC was the most common, with Hap-frequencies of 24.1%. Haplotype GGT was the most uncommon in CD case (3.9%) and control (3.3%) subjects, but occurred at equal frequencies in both (Hap-frequencies of 3.6%, *P* > .05). Haplotypes GAC, GAT, and TGC occurred at equal frequencies in CD patients and control subjects (*P* > .05).

There were also significant associations between these haplotypes for UC cases as compared with control subjects (*χ* ^2^ = 46.3, df = 7, *P* < .0001). For the three marker haplotypes (rs1061622, rs1061624, and rs3397), the frequencies of haplotypes of GGC (9.1% versus 3.1%, *P* < .0001), TAC (17.9% versus 27.7%, *P* = .005), TAT (17.0% versus 6.4%, *P* = .006), and TGT (11.2% versus 23.6%, *P* = .0007) were significantly different between case and control subjects. Haplotype TAC was the most common in UC cases (Hap-frequency of 27.8%, *P* = .005), whereas haplotype GGT was the most uncommon in UC patients and control subjects, with frequencies of 3.6% and 3.3%, respectively (Hap-frequency = 3.4%, *P* = .53). The frequencies of GAC and TGC were lower and the frequency of GAT higher in UC patients than control subjects, but there was no statistically significant difference between them.

## 4. Discussion

The present results show that carrying a single variant allele in TNFRSF1B c.676T > C, 1663A > G, or 1690T > C has no significant effect on the overall risk of either CD or UC. However, the risk of certain phenotypes is affected by either of the latter two variant alleles. More striking, however, is the effect of carrying certain haplotypes, and specific combinations may either increase or decrease the risk of either CD or UC in the New Zealand Caucasian population, with strong statistical significance.

TNFRSF1B is the main TNF receptor found on circulating T cells [14]. The gene contains 10 exons, spanning around 26 kb of genomic DNA and encoding 415 amino acids [14]. Cysteine-rich extracellular regions, comprising three to six disulfide-linked domains, are considered to be important for ligand binding and are expressed by several different cell types [29]. The protein produced by the gene forms a heterocomplex with the product of TNFRSF1A, leading to recruitment of two antiapoptotic proteins c-IAP1 and c-IAP2, both of which possess E3 ubiquitin ligase activity. c-IAP1 has been suggested to potentiate TNF-a induced apoptosis through the ubiquitination and degradation of TNF-receptor-associated factor 2, which produces antiapoptotic signals. We have previously shown that TNF-alpha polymorphisms may contribute significantly to the risk of certain IBD phenotypes in the New Zealand population [9]. The present data augment the previous conclusion, that variant alleles associated with this pathway are important for IBD risk in this population.

There are limited data on the functionality of the various SNPs in this gene. However, the 676 T > G polymorphism replaces a methionine by an arginine, and is functional, as the cytotoxic activity is increased [30]. The variant polymorphism has been previously associated with susceptibility to familial rheumatoid arthritis (RA) [20] and systemic lupus erythematosus (SLE) [21]. However, association studies have suggested that the other SNPs considered in the present study might be in linkage with a functional SNP, even if not themselves functional [22, 23, 31]. Perhaps even more relevant to patients with IBD is that associations have recently been demonstrated between a two allele haplotypes and responses to IFB in Japanese CD patients [28, 32].

Kawasaki et al. [31] reported no association between the variant SNP at position 676 and the risk of either form of IBD in their Japanese database. These data are consistent with our own findings in the present study. However, these data do not agree with other observations in a European Caucasian population [23] which associated TNFRSF1B with clinical CD phenotypes. They studied a well-phenotyped cohort of 205 patients with CD and 106 controls to associate the variant SNP at this position with increased risk of CD (*P* = .034), but decreased risk with UC or with the need for surgery. We suggest that the previous European consortium data had insufficient power to conclusively decide a negative result.

It is of some interest that we have associated differences in the risk of certain IBD phenotypes with variant alleles in the other two SNPs considered (1663A > G, or 1690T > C). A Japanese study considered 124 patients with CD, 106 patients with UC, and 111 unrelated healthy controls [22] for these same two SNPs, while Waschke et al. [23] studied the first but not the second in their European Caucasian group. Neither of these previous reports demonstrated a statistically significant difference in carrying either of the variant alleles. We note, however, that in the Japanese population but not in European groups [23], C appears to be the major allele at position 1493.

Although Sashio et al. [22] reported only minor effects on IBD risk associated with carrying any single variant allele, they found a significant difference (odds ratio of 2.13) in carrier frequency for haplotype AT (1466A, 1493T) between CD patients and the controls. These differences were most significant in CD patients with either internal or external fistula, and/or who responded poorly to nutritional, medical, or surgical therapy. Similar haplotype effects are recorded in the present study. However, we are currently unable to find a convincing explanation of the converse data for UC.

Although the region containing this gene has not emerged from recent whole genome association studies as a lead candidate in CD (WTCCC, 2007; Barrett et al. 2008) it is of interest that two of the genes consistently appearing important are closely related to the present gene. The protein product of TNFAIP3 (TNF-alpha- induced protein 3) inhibits TNF-alpha-induced NFkB-dependent gene expression by interfering with transactivation signals. Markers with lower levels of significance in these studies include one within TNFSF15 (tumour necrosis factor superfamily, member 15). Taken together, the present data add support to an important role for TNF-alpha and related molecules in CD susceptibility and progression.

## Figures and Tables

**Table 1:** TNFRSF1B polymorphisms tested in the Canterbury population.

rs number	Location	Nucleotide change	Amino acid position	Major allele in Europeans	Frequency in other European groups
rs.1061622	*Exon 6 (nonsynonymous)*	c.676T > G	196	T	*75*% *[Hapmap] 80.2*% *[Wachke] *
rs.1061624	*Exon 10 3*′*UTR*	c.*1663A > G		A	*51.7*% *[Hapmap] 55.2*% *[Washke] *
rs.3397	*Exon 10 3*′*UTR*	c.*1690T > C		T	*52.6*% *[Hapmap] *

**Table 2:** Summary of clinical and demographic data for the set of Caucasian IBD patients.

		CD *n* (%)	UC *n* (%)	IC *n* (%)
Gender				
* *Female		249 (64.2)	214 (52.8)	15 (55.6)
* *Male		139 (35.8)	191 (47.2)	12 (44.4)
Age at first diagnosis				
Below 17		39 (10.0)	26 (6.4)	0
Between 17 and 40		199 (51.3)	184 (45.4)	15 (55.6)
Above 40		150 (38.7)	195 (48.2)	12 (44.4)
CD location				
Ileal		125 (32.2)		
Colonic		169 (43.6)		
Ileocolonic		90 (23.2)		
Unknown (U + UN)		4 (1.0)		
UC location				
Proctitis			140 (34.6)	3 (11.1)
Left colon			107 (26.4)	5 (18.5)
Pancolitis			154 (38.0)	19 (70.4)
Unknown			4 (1.0)	0
Behaviour				
Nonstricturing, nonpenetrating perianal disease:	With	47 (21.5)		
	Without	172 (78.5)		
Stricturing perianal disease:	With	46 (38.0)		
	Without	75 (62.0)		
Penetrating perianal disease:	With	17 (35.4)		
	Without	31 (64.6)		
Any relative with IBD: Yes (*n* = 143)		74 (19.1)	65 (16.1)	5 (18.5)
Bowel resection: Yes (*n* = 214)		142 (36.6)	70 (17.3)	2 (7.4)
Smoker at diagnosis: Yes (*n* = 147)		97 (25.7)	49 (12.3)	2 (7.7)
Ever used immunomodulators: Yes (*n* = 296)		203 (52.3)	86 (21.2)	8 (29.6)
Extraintestinal manifestations: Yes (*n* = 142)		75 (19.3)	64 (15.8)	3 (11.1)

**Table 3:** Primer and probe sequences for custom-made TaqMan SNP genotyping assay for TNFRSF1B.

TaqMan SNP genotyping assay	DNA sequence
TNFRSF1B_*1663A > G forward primer	5′ TGACCTGCAGGCCAAGAG 3′
TNFRSF1B_*1663A > G_reverse primer	5′ CCATGGCAGCAGAGGCTTT 3′
TNFRSF1B_*1663A > G_VIC probe	5′ CCACAACTCGCTGCC 3′
TNFRSF1B_*1663A > G_FAM probe	5′ CACAACCCGCTGCC 3′
TNFRSF1B_*1690T > C_forward primer	5′ TGACCTGCAGGCCAAGAG 3′
TNFRSF1B_*1690T > C_reverse primer	5′ CCATGGCAGCAGAGGCTTT 3′
TNFRSF1B_*1690T > C_VIC probe	5′ CATGGCGTGTCCC 3′
TNFRSF1B_*1690T > C_FAM probe	5′ CCATGGTGTGTCCC 3′

**Table 4:** Genotype and allele counts for TNFRSF1B variants in New Zealand IBD patients and in New Zealand Caucasians.

SNP		Controls	CD		UC		CD + UC		IC
		N(%)	N(%)	OR (95% CI)	N(%)	OR (95% CI)	N(%)	OR (95% CI)	N(%)
TNFRSF1B_1061622	GG	12 (4.1)	18 (4.7)	1.12 (0.55–2.28)	23 (5.8)	1.42 (0.72–2.79)	41 (5.2)	1.27 (0.69–2.34)	0
	GT	102 (34.8)	144 (37.6)	1.17 (0.86–1.59)	154 (38.6)	1.22 (0.90–1.65)	298 (38.1)	1.19 (0.91–1.56)	8 (30.8)
	TT	179 (61.1)	221 (57.7)	1.00	222 (55.6)	1.00	443 (56.6)	1.00	18 (69.2)
Genotype *P*-value			0.67		0.29		0.38		
HWE *P*-value		.73	.37		.58		.32		
	G	126 (21.5)	180 (23.5)		200 (25.1)		380 (24.3)		44 (84.6)
	T	460 (78.5)	586 (76.5)		598 (74.9)		1184 (75.7)		8 (15.4)
OR (95% CI)			1.12 (0.87–1.45)		1.22 (0.95–1.57)		1.17 (0.93–1.47)		
Allelic *P*-value			.38		.12		.17		
TNFRSF1B_1061624	AA	65 (22.2)	94 (24.5)	1.00	96 (24.1)	1.00	190 (24.3)	1.00	4 (16.0)
	AG	158 (53.9)	185 (48.3)	0.85 (0.59–1.22)	185 (46.4)	0.84 (0.59–1.21)	370 (47.3)	0.85 (0.62–1.16)	20 (80.0)
	GG	70 (23.9)	104 (27.2)	1.05 (0.69–1.59)	118 (23.9)	1.21 (0.80–1.82)	222 (28.4)	1.13 (0.79–1.63)	1 (4.0)
Genotype *P*-value			0.43		0.12		0.16		
HWE *P*-value		.20	.51		.16		.14		
	A	288 (49.1)	373 (48.7)		377 (47.2)		750 (48.0)		28 (56.0)
	G	298 (50.9)	393 (51.3)		421 (52.8)		814 (52.0)		22 (44.0)
OR (95% CI)			0.98 (0.79–1.22)		0.93 (0.75–1.15)		0.95 (0.79–1.15)		
Allelic *P*-value			.87		.48		.62		
TNFRSF1B_3397	CC	112 (38.2)	135 (35.2)	1.00	165 (41.4)	1.00	300 (38.4)	1.00	11 942.3)
	CT	138 (47.1)	186 (48.6)	1.22 (0.89–1.67)	169 (42.4)	0.88 (0.65–1.21)	355 (45.4)	1.03 (0.78–1.36)	11 (42.3)
	TT	43 (14.7)	62 (16.2)	1.29 (0.83–2.02)	65 (16.3)	1.09 (0.71–1.68)	127 (16.2)	1.18 (0.80–1.74)	4 (15.4)
Genotype *P*-value			.70		.46		.79		
HWE *P*-value		1.00	0.88		0.05		0.20		
	C	362 (61.8)	456 (59.5)		499 (62.5)		955 (61.1)		33 (63.5)
	T	224 (38.2)	310 (40.5)		299 (37.5)		609 (38.9)		19 (36.5)
OR (95% CI)			0.91 (0.73–1.13)		1.03 (0.83–1.29)		0.97 (0.80–1.18)		
Allelic *P*-value			.40		.77		.76		

**Table 5:** Allelic odds ratios and 95% confidence intervals for comparison of TNFRSF1B variants with IBD status in New Zealand IBD patients and Caucasians.

	Allelic odds ratios and 95%				Allelic odds ratios and 95%				Allelic odds ratios and 95%			
	confidence intervals for comparison of				confidence intervals for comparison of				confidence intervals for comparison of			
	TNFRSF1_B1061622				TNFRSF1B_1061624				TNFRSF1B_3397			
	Crohns disease		Ulcerative colitis		Crohns disease		Ulcerative colitis		Crohns disease		Ulcerative colitis	
	OR	95% CI	OR	95% CI	OR	95% CI	OR	95% CI	OR	95% CI	OR	95% CI
Female	1.20	0.86–1.67	1.38	0.99–1.93	1.10	0.84–1.44	1.00	0.76–1.32	0.85	0.65–1.12	0.96	0.72–1.27
Male	1.05	0.72–1.54	1.02	0.72–1.45	0.82	0.59–1.14	0.79	0.59–1.07	0.89	0.64–1.24	1.04	0.76–1.42
Age at first diagnosis												
0–16 years	0.85	0.47–1.54	1.08	0.55–2.11	0.88	0.55–1.42	1.30	0.74–2.29	0.86	0.53–1.39	1.22	0.67–2.22
17–40 years	1.26	0.94–1.68	1.18	0.87–1.59	1.01	0.79–1.29	0.93	0.72–1.20	0.85	0.66–1.09	0.96	0.74–1.25
>40 years	1.01	0.73–1.41	1.24	0.93–1.66	0.95	0.72–1.25	0.83	0.65–1.07	0.89	0.67–1.17	1.00	0.77–1.29
CD location												
Ileal	0.91	0.63–1.30			1.13	0.85–1.51			0.88	0.65–1.18		
Colonic	1.17	0.86–1.59			0.90	0.69–1.17			0.91	0.70–1.19		
Ileocolonic	1.27	0.87–1.86			0.90	0.64–1.26			0.78	0.56–1.09		
UC location												
Pancolitis			1.13	0.82–1.56			0.94	0.72–1.23			1.14	0.86–1.51
Left colon			1.24	0.87–1.78			**0.73**	0.54–1.00			0.92	0.67–1.26
Proctitis			1.27	0.92–1.76			1.00	0.76–1.32			0.93	0.70–1.24
CD Behavior												
Inflammatory	1.17	0.88–1.56			0.88	0.69–1.12			0.92	0.72–1.18		
Stricturing	1.03	0.72–1.47			1.01	0.75–1.36			0.90	0.67–1.22		
Penetrating	1.13	0.68–1.87			1.38	0.90–2.12			**0.62**	**0.40**–**0.95**		
Ileal/stricturing	0.86	0.55–1.34			1.03	0.72–1.46			0.93	0.65–1.33		
Colonic/inflammatory	1.24	0.90–1.71			0.84	0.64–1.11			0.93	0.70–1.23		
Any relative with IBD	1.22	0.80–1.86	1.15	0.74–1.79	0.78	0.54–1.12	0.88	0.60–1.28	0.97	0.67–1.40	0.83	0.57–1.22
Bowel resection	0.93	0.66–1.31	1.10	0.71–1.70	1.09	0.83–1.44	0.79	0.55–1.14	0.77	0.58–1.02	1.29	0.87–1.90
Smoker at diagnosis	0.96	0.65–1.42	1.16	0.71–1.90	1.01	0.73–1.39	**0.62**	**0.40–0.96**	0.92	0.66–1.28	1.22	0.78–1.91
Ever used immunomodulators	1.08	0.80–1.45	1.43	0.98–2.09	1.07	0.84–1.37	1.16	0.83–1.62	0.82	0.64–1.05	0.88	0.63–1.24
Any EIMs	1.05	0.68–1.61	1.05	0.66–1.66	1.06	0.74–1.51	0.83	0.57–1.21	0.89	0.62–1.28	0.81	0.55–1.19

**Table 6:** Haplotype analysis of three-SNP TNFRSF1B haplotype in IBD status in New Zealand IBD patients and Caucasians.

	Haplotype	Case frequency	Control frequency	Hap frequency	Hap-score	*P*-value	Global score statistics
CD	Three SNPs TNFRSF1B region (rs1061622, rs1061624, rs3397)						
	GAC	0.057	0.101	0.075	−1.55	.12	*χ* ^2^ = 29.9, df = 7, *P* = .0001
	GAT	0.048	0.050	0.051	0.09	.93	
	GGC	0.091	0.031	0.064	2.78	.005	
	GGT	0.039	0.033	0.036	0.77	.44	
	TAC	0.210	0.277	0.241	−1.88	.06	
	TAT	0.172	0.064	0.122	3.29	.001	
	TGC	0.238	0.209	0.224	0.16	.88	
	TGT	0.145	0.236	0.186	−1.90	.06	
UC	Three SNPs TNFRSF1B region (rs1061622, rs1061624, rs3397)						
	GAC	0.067	0.101	0.078	−0.92	.36	*χ* ^2^ = 46.3, df = 7, *P* < .0001
	GAT	0.057	0.050	0.056	0.43	.67	
	GGC	0.091	0.031	0.067	3.32	<.0001	
	GGT	0.036	0.033	0.034	0.63	.53	
	TAC	0.179	0.277	0.278	−2.81	.005	
	TAT	0.170	0.064	0.128	2.76	.006	
	TGC	0.289	0.209	0.259	1.75	.08	
	TGT	0.112	0.236	0.159	−3.40	.0007	
